# Neohesperidin enhances PGC-1α-mediated mitochondrial biogenesis and alleviates hepatic steatosis in high fat diet fed mice

**DOI:** 10.1038/s41387-020-00130-3

**Published:** 2020-08-05

**Authors:** Si-wei Wang, Hao Sheng, Yong-feng Bai, Yuan-yuan Weng, Xue-yu Fan, Li-jun Lou, Feng Zhang

**Affiliations:** 1 Department of Core Facility, The People’s Hospital of Quzhou, 324000 Quzhou, China; 2 Department of Pharmacy, The People’s Hospital of Quzhou, 324000 Quzhou, China; 3 Zhejiang University School of Medicine, 310058 Hangzhou, China; 4 Department of Clinical Laboratory, The People’s Hospital of Quzhou, 324000 Quzhou, China

**Keywords:** Type 2 diabetes, Translational research

## Abstract

**Backgrounds:**

Mitochondria plays a critical role in the development and pathogenesis of nonalcoholic fatty liver disease (NAFLD). Neohesperidin (NHP) could lower blood glucose and prevent obesity in mice. However, the direct effect of NHP on hepatic steatosis has not been reported.

**Methods:**

Mice were fed with either a chow diet or HFD with or without oral gavage of NHP for 12 weeks. A variety of biochemical and histological indicators were examined. In vitro cell culture model was utilized to demonstrate underlying molecular mechanism of the effect induced by NHP treatment.

**Results:**

NHP increases mitochondrial biogenesis, improves hepatic steatosis and systematic insulin resistance in high fat diet (HFD) fed mice. NHP elevates hepatic mitochondrial biogenesis and fatty acid oxidation by increasing PGC-1α expression. Mechanistically, the activation of AMP-activated protein kinase (AMPK) is involved in NHP induced PGC-1α expression.

**Conclusions:**

PGC-1α-mediated mitochondrial biogenesis plays a vital role in the mitigation of hepatic steatosis treated by NHP. Our result suggests that NHP is a good candidate to be dietary supplement for the auxiliary treatment of NAFLD.

## Introduction

Hypercaloric diet is one of the main factors leading to metabolic syndrome, mainly manifested as obesity, hyperlipidemia, insulin resistance and fatty liver, among others^1^. Nonalcoholic fatty liver disease (NAFLD), which is characterized by the accumulation of excess fat in the liver of people who drink little or no alcohol^2^, is increasingly recognized as the hepatic manifestation of metabolic syndrome. The progression of NAFLD leads to liver cirrhosis and liver cancer^1,3^. However, the treatment for NAFLD is still limited.

It has been known that mitochondria play a critical role in the development and pathogenesis of NAFLD^4,5^. Mitochondrial biogenesis is essential to augment mitochondrial capacity, which helps relieving lipid accumulation in liver^6^. Proliferator-activated receptor γ coactiva-tor-1α (PGC-1α) is a key regulator of energy homeostasis by transcriptional regulation of genes involved in fatty acid oxidation and mitochondrial biology^5,7,8^. Previous researches have shown a 40% decrease in hepatic PGC-1α expression in NAFLD patients, accompanied by mitochondrial dysfunction, lipid accumulation, and insulin resistance^9,10^. Furthermore, the treatment that stimulates mitochondrial function can delay the progression of obesity and diabetes^5^. Therefore, PGC-1α-mediated mitochondrial biogenesis is essential for the improvement of NAFLD.

Traditional medicine and complementary have gained more attention for long-term use in treating metabolic diseases like obesity and diabetes due to less side-effects compared with synthetic chemical drugs^11,12^. Clinical studies reveal that dietary intake of flavonoids can reduce the risk of NAFLD^13,14^. Our previous study has shown that Quzhou Fructus Aurantii, which is rich in flavonoids, could ameliorate fatty liver and insulin resistance in high-fat diet (HFD) fed mice^15^. However, its mechanism is still unclear. Neohesperidin is the main component of Quzhou Fructus Aurantii^15^. It is reported that neohesperidin could inhibit lipid accumulation in adipocytes, lowers blood glucose and lipid and prevent obesity in mice^16,17^. However, the direct effect of neohesperidin on hepatic steatosis has not been reported.

In current study, we found that NHP elevated PGC-1α expression and hepatic mitochondrial biogenesis in HFD fed mice. It also enhanced fatty acid oxidation, alleviated hepatic steatosis and insulin resistance. Mechanistically, NHP induced AMPK activation is involved in this process.

## Materials and methods

The research reported here was approved by the Ethics Committee of Quzhou People’s Hospital. All methods were carried out in accordance with the relevant guidelines and regulations.

### Animal experiments

The protocol of animal experiment was approved by the Ethics Committee of Animal Experiments of Quzhou people’s hospital, China. Animal experiment was conducted at the Experimental Animal Center of Zhejiang University of Traditional Chinese Medicine, China. Eight-week-old male C57BL/6 male were purchased from GemPharmatech Co., Ltd., Jiangsu, China (license number of animal production: SYXK 2015-0001). All animals were kept under standard conditions with having free access to distilled water and common pelleted food. After one week of acclimation, the mice were randomly distributed into 3 groups of 12 mice: chow group, which were fed a chow diet (Provided by the Experimental Animal Center of Zhejiang University of Traditional Chinese Medicine) and received intragastrically administered distilled water; HFD group, which were fed a high fat diet with 60 kcal% fat (Research diet D12492, Research Diet, NJ) and received intragastrically administered distilled water; HFD + NHP group, which were fed HFD and treated with 50 mg/kg per day of intragastrically administered neohesperidin (NHP, CAS# 13241-33-3, Shanghai Yuanye Biological Technology, China) for 12 weeks. Food intake, body weight and fasting blood glucose (FBG) were measured once a week.

### Biochemical testing

Fasting blood glucose (FBG) levels were measured by blood glucose meter (Johnson & Johnson, USA). Serum alanine aminotransferase (ALT), aspartate aminotransferase (AST), triglyceride (TG), nonesterified fatty acid (NEFA), total cholesterol (TC) levels were detected by biochemical analyzer according to the manufacturer’s instruction (DiaSys Diagnostic Systems, Shanghai, China). Hepatic triglyceride (TG) and cholesterol (TC) contents were assessed using enzymatic reactions with commercial kits (Dongou Diagnostics Co., LTD, Zhejiang, China). Hepatic malondialdehyde (MDA), reactive oxygen species (ROS), superoxide dismutase (SOD), catalase (CAT), reduced glutathione (GSH) were tested by using commercially available kits according to the manufacturer’s instructions (Nanjing Jiancheng Bioengineering Institute, China).

### Oral glucose tolerance test (OGTT) and insulin tolerance test (ITT)

At the 8th week of the experiment, 5 mice were taken from each group for the OGTT and ITT. For OGTT, the mice were fasted for 12 h and then oral d-glucose (2 g/kg). For ITT, the mice were fasted for 6 h and then injected i.p. with insulin (0.75 U/kg). Blood glucose levels were measured at 0, 30, 60, 90, and 120 min, which was measured by tail vein using a standard glucometer (Johnson & Johnson, USA).

### Histopathological analysis

Liver tissues were fixed in 10% formalin and processed into paraffin sections. Then the sections were stained with hematoxylin and eosin (H&E) staining. NAFLD activity score (NAS) was scored in a blinded manner according to Kleiner et al.^18^ Macrovesicular steatosis: score 0: <5% (minimal), score 1: 5–33% (mild), score 2: 34–66% (moderate), score 3: >66% (severe); lobular inflammation: score 0: none, score 1: <2 foci/200× field, score 2: 2–4 foci/200× field, score 3: >4 foci/200× field; hepatocellular ballooning: score 0: none, score 1: few, score 2: many. These three values were then added to obtain the NAS score (range 0–8). Mean scores were evaluated through calculating five different 200× microscopic fields per mouse section by two independent trained observers.

Liver tissues were processed into frozen sections. The sections were stained with oil red O staining from each group according to the manufacturer’s instructions (Solarbio Life Science, China) and the intensity of Oil Red O was quantified with ImageJ software (U.S. National Institutes of Health, Bethesda, MD). The final count represented the mean of percentage of stained area from five randomly selected 400× microscopic fields per mouse section.

### Immunohistochemical staining

The liver tissues sections were deparrafinized and antigen retrieval were also performed as previously described^19^. MaxVision HRP-Polymer antiRabbit IHC Kit (MXB Biotechnologies, Fuzhou, China) was used to develop signal. The antibody used in this part was antiMPO (1: 50, ab9535, Abcam). The number of MPO-positive cells was calculated in 5 randomly selected 200× microscopic fields per mouse section using ImageJ software (U.S. National Institutes of Health, Bethesda, MD).

### Cell culture and treatment

Human hepatoma HepG2 cell line was obtained from the Shanghai Bank of Cell Lines (Shanghai, China) and cultured in Dulbecco’s Modified Eagle’s Medium (DMEM) containing 10% fetal bovine serum (FBS, BBI Life Sciences Corporation, China), 100 U/mL penicillin, and 100 U/mL streptomycin at 37 °C in a humidified atmosphere with 5% CO_2_. The source of the cell line was Identified by STR profiling and tested for mycoplasma contamination. HepG2 cells were intervened by 0.4 mM palmitate (PA) after starving in serum-free DMEM for 24 h to establish the hepatic steatosis model. The cells were treated with DMSO, 0.4 mM PA, 0.4 mM PA + 100 μM NHP, 0.4 mM PA + 100 μM NHP + 20 μM SR-18292 (CAS#2095432-55-4, Sigma-Aldrich, USA) or 0.4 mM PA + 100 μM NHP + 100 nM Compound C (CAS#866405-64-3, Merch Millipore, USA) for 16 h, respectively. Then the cells were lysed, RNA and protein were collected for further measurement.

### Mitochondrial function assay

Succinate dehydrogenase activity was detected by using 3-(4,5-dimethyl-2-thiazolyl)-2,5-diphenyl-2-H-tetrazolium bromide (MTT) assay, as our previously described^20^. ATP content was measured with ATP Determination Kit (Molecular Probes) according to the manufacturer’s instructions (#A22066, Thermo Fisher Scientific, USA).

### Mitochondrial staining

HepG2 cells were treated with DMSO, 0.4 mM PA, and 0.4 mM PA + 100 μM NHP for 16 h. Then, the cells were stained with 200 nM Mito-Tracker Red (#M7512, ThermoFisher, USA) for 60 min at 37 °C, according to the manufacturer’s instruction. The fluorescence was visualized by a SUNNY RX50 fluorescence microscope. Fluorescence intensity was detected by using Microplate Reader (BioTek, USA) at Ex 579/Em 599 nm.

### Real-time PCR and mtDNA analysis

Total RNA from liver tissues or hepatic cells, was collected using Trizol Reagent (#DP424, Tiangen Biotech Co. Ltd., Beijing, China) according to the instruction of the manufacturer. The cDNA was prepared by Maxima Reverse Transcriptases (#EP0751, Thermo Fisher Scientific, USA). The mitochondria extraction and purification of liver tissue was using QIAamp DNA Mini Kit (#51304, Qiagen) according to the manufacturer’s protocol^21^. Real-time PCR was performed in triplicate using SGExcel FastSYBR Mixture (#B532955-0005, Sangon Biotech Co., Ltd., Shanghai, China) by Roche LightCyclerR 480 Quantitative PCR System (Indianapolis, USA). Normal relative expression analysis was normalized to the internal control Gapdh or β-actin. mtDNA analysis was quantified by qPCR using primers specific for the mitochondrial cytochrome c oxidase subunit 2 (COX2) gene and normalized to genomic DNA by amplification of the ribosomal protein s18 (Rps18) nuclear gene. Primers are listed in Table 1.

**Table 1:** The primers used in this study for real time PCR.

Description	Sense primer (5′→3′)	Antisense primer (5′→3′)
*Srebf1*	CAAGGCCATCGACTACATCCG	CACCACTTCGGGTTTCATGC
*Fasn*	GGAGGTGGTGATAGCCGGTAT	TGGGTAATCCATAGAGCCCAG
*Scd1*	TTCTTGCGATACACTCTGGTGC	CGGGATTGAATGTTCTTGTCGT
*Acc1*	CTCCCGATTCATAATTGGGTCTG	TCGACCTTGTTTTACTAGGTGC
*Pgc-1α*	CAGTCGCAACATGCTCAAG	TGGGGTCATTTGGTGACTCT
*Pparα*	CCTCAGGGTACCACTACGGAGTT	CCGAATAGTTCGCCGAAAGA
*Pdk4*	AGGGAGGTCGAGCTGTTCTC	GGAGTGTTCACTAAGCGGTCA
*Acaa2*	CTGCTACGAGGTGTGTTCATC	AGCTCTGCATGACATTGCCC
*Cpt-1*	TGGCATCATCACTGGTGTGTT	GTCTAGGGTCCGATTGATCTTTG
*Acox1*	GCCAATGCTGGTATCGAAGAA	AATCCCACTGCTGTGAGAATAGC
*Nrf-1*	AGCACGGAGTGACCCAAAC	AGGATGTCCGAGTCATCATAAGA
*Tfam*	AACACCCAGATGCAAAACTTTCA	GACTTGGAGTTAGCTGCTCTTT
*COX-2*	ATAACCGAGTCGTTCTGCCAAT	TTTCAGAGCATTGGCCATAGAA
*Rsp18*	TGTGTTAGGGGACTGGTGGACA	CATCACCCACTTACCCCCAAAA
*Il-6*	CTGCAAGAGACTTCCATCCAG	AGTGGTATAGACAGGTCTGTTGG
*Il-1β*	TTCAGGCAGGCAGTATCACTC	GAAGGTCCACGGGAAAGACAC
*Tnf-α*	CTGAACTTCGGGGTGATCGG	GGCTTGTCACTCGAATTTTGAGA
*Cat*	GGAGGCGGGAACCCAATAG	GTGTGCCATCTCGTCAGTGAA
*Sod1*	AACCAGTTGTGTTGTCAGGAC	CCACCATGTTTCTTAGAGTGAGG
*Gpx1*	CCACCGTGTATGCCTTCTCC	AGAGAGACGCGACATTCTCAAT
*Ucp2*	ATGGTTGGTTTCAAGGCCACA	TTGGCGGTATCCAGAGGGAA
*Gapdh*	TGAGGCCGGTGCTGAGTATGT	CAGTCTTCTGGGTGGCAGTGAT
*PGC-1α*	TCTGAGTCTGTATGGAGTGACAT	CCAAGTCGTTCACATCTAGTTCA
*NRF-1*	AGGAACACGGAGTGACCCAA	TATGCTCGGTGTAAGTAGCCA
*TFAM*	ATGGCGTTTCTCCGAAGCAT	TCCGCCCTATAAGCATCTTGA
*β-ACTIN*	AGAGCTACGAGCTGCCTGAC	AGCACTGTGTTGGCGTACAG

### Western blot analysis

The protein extraction method was according to our previous study^19,22^. Equal amounts of proteins were separated by sodium dodecyl sulfate-polyacrylamide gel electrophoresis (SDS-PAGE). Then, the separated proteins were transferred to polyvinylidene fluoride (PVDF) membrane and blocked with 1% casein at room temperature for 1 h. Subsequently, the membrane incubated overnight at 4 °C with the primary antibodies against antiphospho-AMPKα (1: 1000, #2535, Cell Signaling Technology), antiAMPKα (1: 1000, #5831, Cell Signaling Technology), and antiβ-actin (1:3000, A1978, Millipore Sigma). After washing with TBST for four times, the membranes were incubated with HRP-conjugated secondary antibodies for 1 h at room temperature. Immunoreactive bands were visualized using Tanon 4200SF system (Tanon Biotechnology, Shanghai, China) and quantified densitometry using Image J software (U.S. National Institutes of Health, Bethesda, MD).

### Statistical analysis

All of data are presented as mean ± SD values. Statistical significance was evaluated using Student’s unpaired two-tailed *t*-test and among more than two groups by analysis of one-way ANOVA with Bonferroni’s post hoc test. *p* values of <0.05 were considered statistically significant and *p* values of <0.01 were considered statistically highly significant. Analysis was performed using GraphPad Prism software, version 5.0 (GraphPad Software, La Jolla, CA).

## Results

### NHP improves liver function in HFD-fed mice

**Fig. 1:**
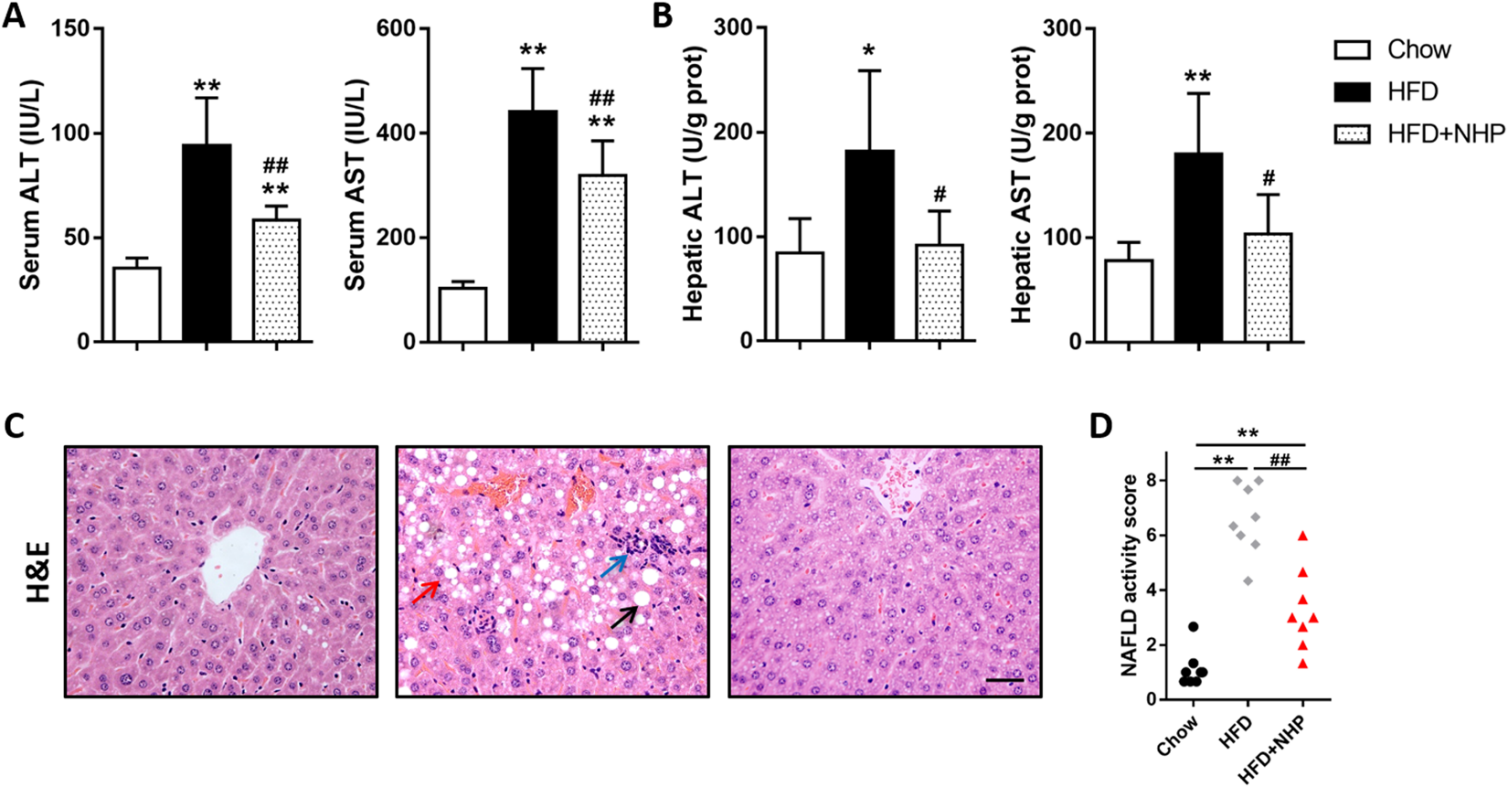
The effect of NHP administration on liver function in HFD-fed mice. C57BL/6 mice were fed either a chow or a high fat diet (HFD) for 12 weeks. Mice were treated with daily oral doses of NHP (50 mg/kg). Water was gavaged as control. **a** Serum alanine aminotransferase (ALT) and aspartate aminotransferase (AST) levels. **b** Hepatic alanine aminotransferase (ALT) and aspartate aminotransferase (AST) levels (*n* = 12). **c** The representative images of H&E staining in livers from each group. Scale bar = 300 μm. Black arrow represents macrovesicular steatosis; red arrow represents hepatocellular ballooning; blue arrow represents lobular inflammation. **d** The NAFLD activity score was shown. Data were expressed as the mean ± SD (*n* = 12). *p < 0.05, **p < 0.01, versus chow group; ^#^*p* < 0.05, ^##^*p* < 0.01, versus HFD group.

Long-term feeding of high-fat diet can cause hepatic lipid accumulation and impaired liver function. Compared to chow diet fed mice, the HFD mice displayed evidently more body weight gain and adipose tissue weight (Fig. S1). Administration of NHP reduced body weight gain significantly and adipose tissue weight to some extent, but had no effect on food intake in HFD-fed mice (Fig. S1). Meanwhile, NHP treatment decreased serum and liver ALT and AST levels in mice fed with HFD (Fig. 1a, b), suggesting improved liver function. In addition, administration of NHP obviously relieved hepatic steatosis in HFD mice indicated by H&E staining (Fig. 1c) and NAFLD activity score (NAS) evaluation (Fig. 1d).

### NHP inhibits hepatic lipid accumulation in HFD-induced mice

**Fig. 2:**
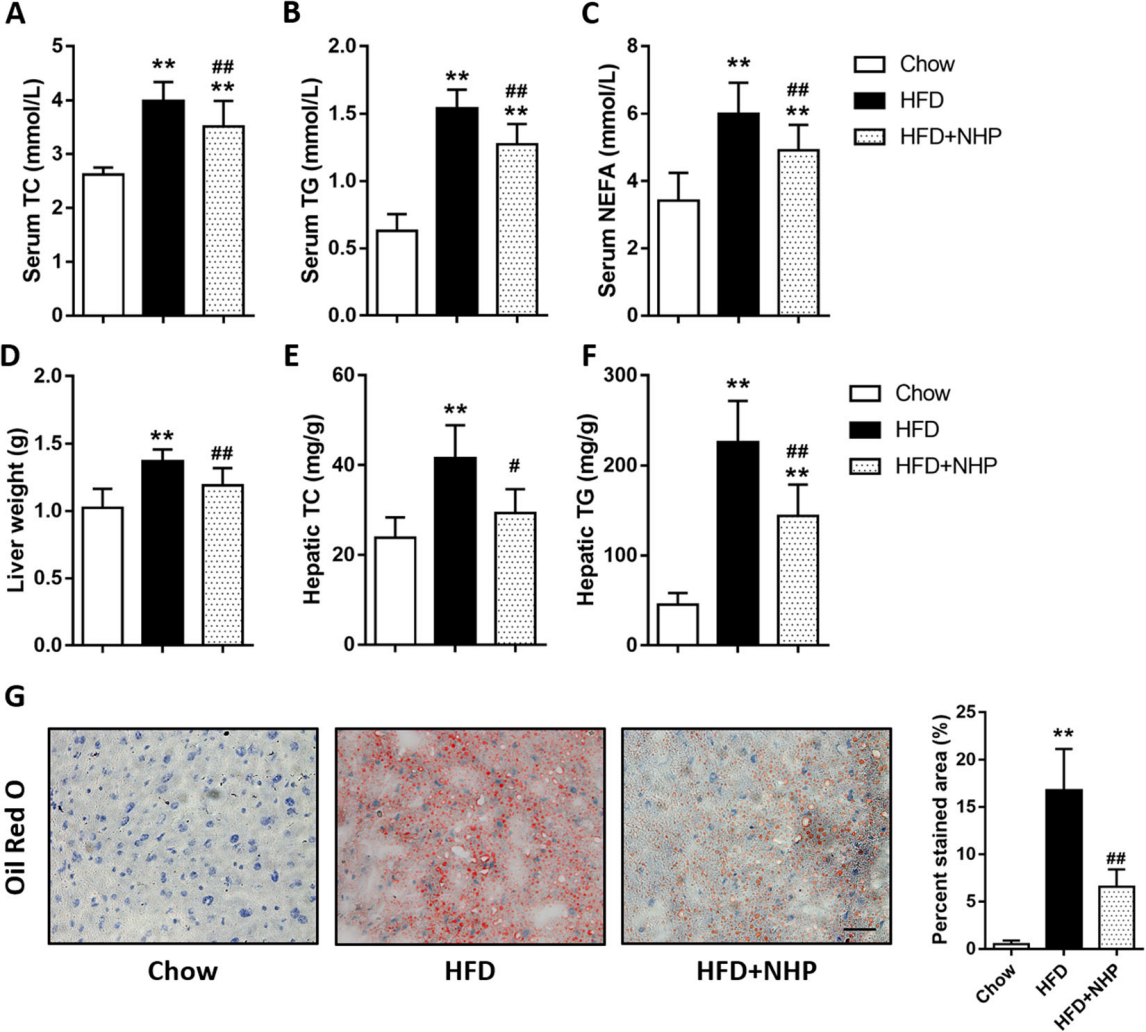
NHP administration reduces the blood and liver lipid levels in HFD-induced mice. **a** Serum total cholesterol (TC) level. **b** Serum triglyceride (TG) level. **c** Serum nonesterified fatty acid (NEFA) level. **d** Liver weight (g). **e** Hepatic total cholesterol (TC) level. **f** Hepatic triglyceride (TG) level. Data were expressed as the mean ± SD (*n* = 12). **g** The representative images of Oil Red O staining in livers from each group. Scale bar = 300 μm. The quantification of Oil Red O-stained areas was shown. Data were expressed as the mean ± SD (*n* = 12). *p < 0.05, **p < 0.01, versus chow group; ^#^*p* < 0.05, ^##^*p* < 0.01, versus HFD group.

To further characterize the effect of NHP on hepatic pathophysiology in HFD fed mice, we assessed serum and hepatic indicators for lipid metabolism. NHP treatment reduced TC, TG, and NEFA levels in serum (Fig. 2a, c). Moreover, hepatic TC and TG as well as liver weight were decreased upon NHP administration (Fig. 2d, f). The hepatic lipid accumulation examined by Oil Red O staining revealed that the percentage of stained area reduced to nearly the half by NHP in HFD mice (Fig. 2g).

Insulin resistance is one of the most important features in HFD-induced mice^15^. NHP treatment obviously improved the levels of fasting blood glucose (FBG), fasting serum insulin (FINS) and HOMA-IR in HFD fed mice (Fig. S2A to C). The results from oral glucose-tolerance tests (OGTT) and insulin-tolerance tests (ITT) showed that NHP also markedly improved peripheral insulin resistance and glucose intolerance in HFD mice (Fig. S2D and E).

### NHP relieves hepatic inflammation and oxidative stress in HFD-induced mice

**Fig. 3:**
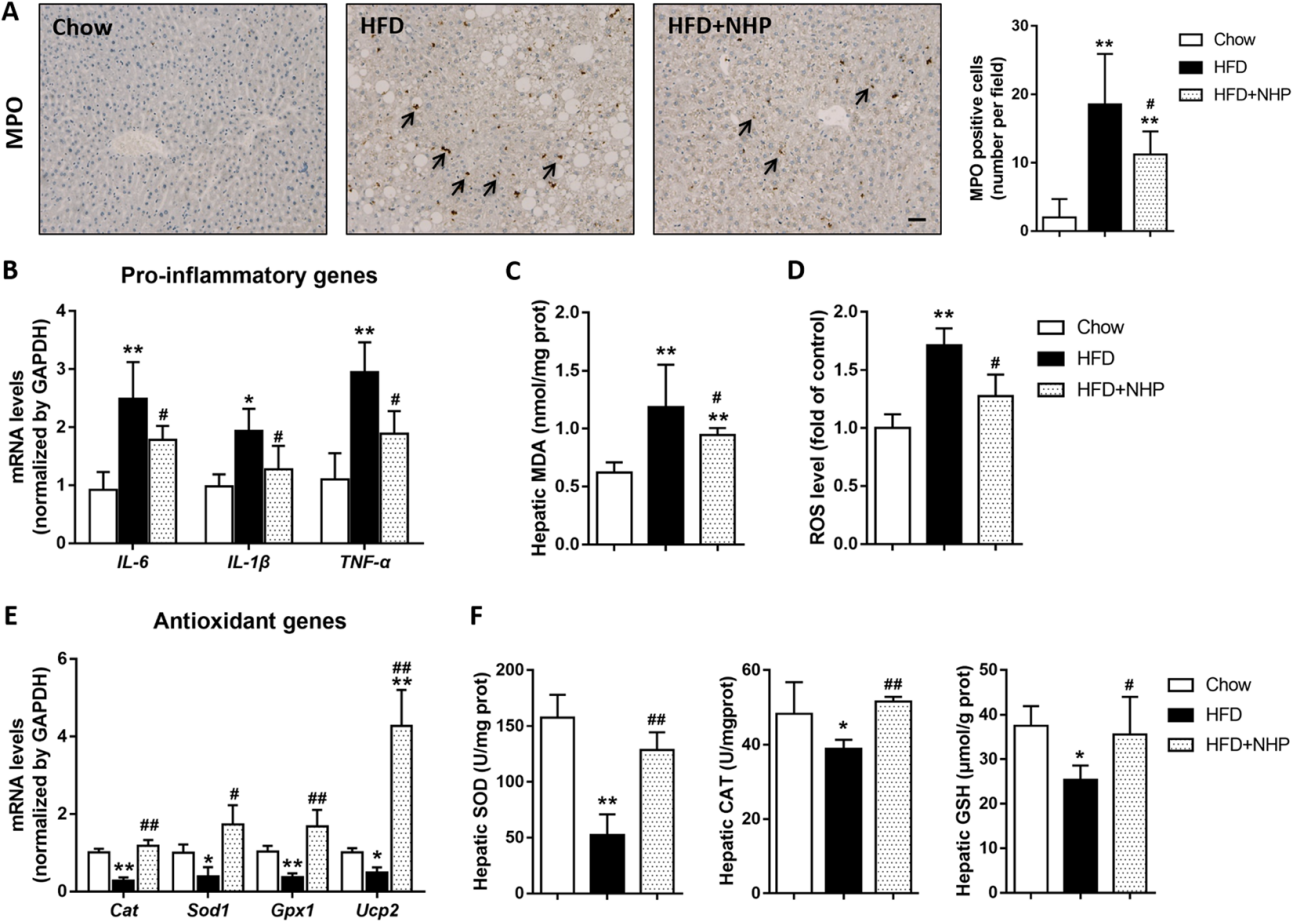
NHP relieves hepatic inflammation and oxidative stress in liver of HFD-induced mice. **a** The representative images of myeloperoxidase (MPO) immunohistochemical staining in liver from each group. Scale bar = 300 μm. Black arrow points to MPO-positive cells. Quantification and statistical analysis of MPO-positive cells in liver from each group. Values were expressed as mean ± SD (*n* = 12). **b** The mRNA expressions of *Il-6*, *Il-1β* and *Tnf-α* were determined by RT-PCR. Values were expressed as mean ± SD (*n* = 5). **c** Hepatic malondialdehyde (MDA) level. **d** Hepatic reactive oxygen species (ROS) level. Data were expressed as the mean ± SD (*n* = 12). **e** The mRNA expressions of *Cat*, *Sod1*, *Gpx1,* and *Ucp2* were determined by RT-PCR. Values were expressed as mean ± SD (*n* = 5). **f** Hepatic superoxide dismutase (SOD) activity, catalase (CAT) activity and reduced glutathione (GSH) level in each group. Data were expressed as the mean ± SD (*n* = 12). *p < 0.05, **p < 0.01, versus chow group; ^#^*p* < 0.05, ^##^*p* < 0.01, versus HFD group.

Long-term excessive lipid accumulation in liver can cause inflammatory response and oxidative stress^23,24^. Our results showed that the number of MPO positive neutrophils were less in NHP treated group compared to their HFD counterparts (Fig. 3a). The mRNA expression of inflammatory factors such as *Il-6*, *Il-1β*, *Tnf-α* were also reduced by NHP treatment (Fig. 3b). NHP treatment significantly reduced hepatic MDA and ROS level, markers of oxidative stress (Fig. 3c, d). Additionally, the mRNA expression of antioxidant genes *Cat*, *Sod1*, *Gpx1,* and *Ucp2* was increased by NHP treatment (Fig. 3e). At the same time, the levels of intracellular antioxidant enzymes (SOD, CAT) and ROS scavenger (GSH) were also increased by NHP in the liver of HFD induced mice (Fig. 3f).

### NHP suppresses fatty acid synthesis and promotes fatty acid oxidation in liver

**Fig. 4:**
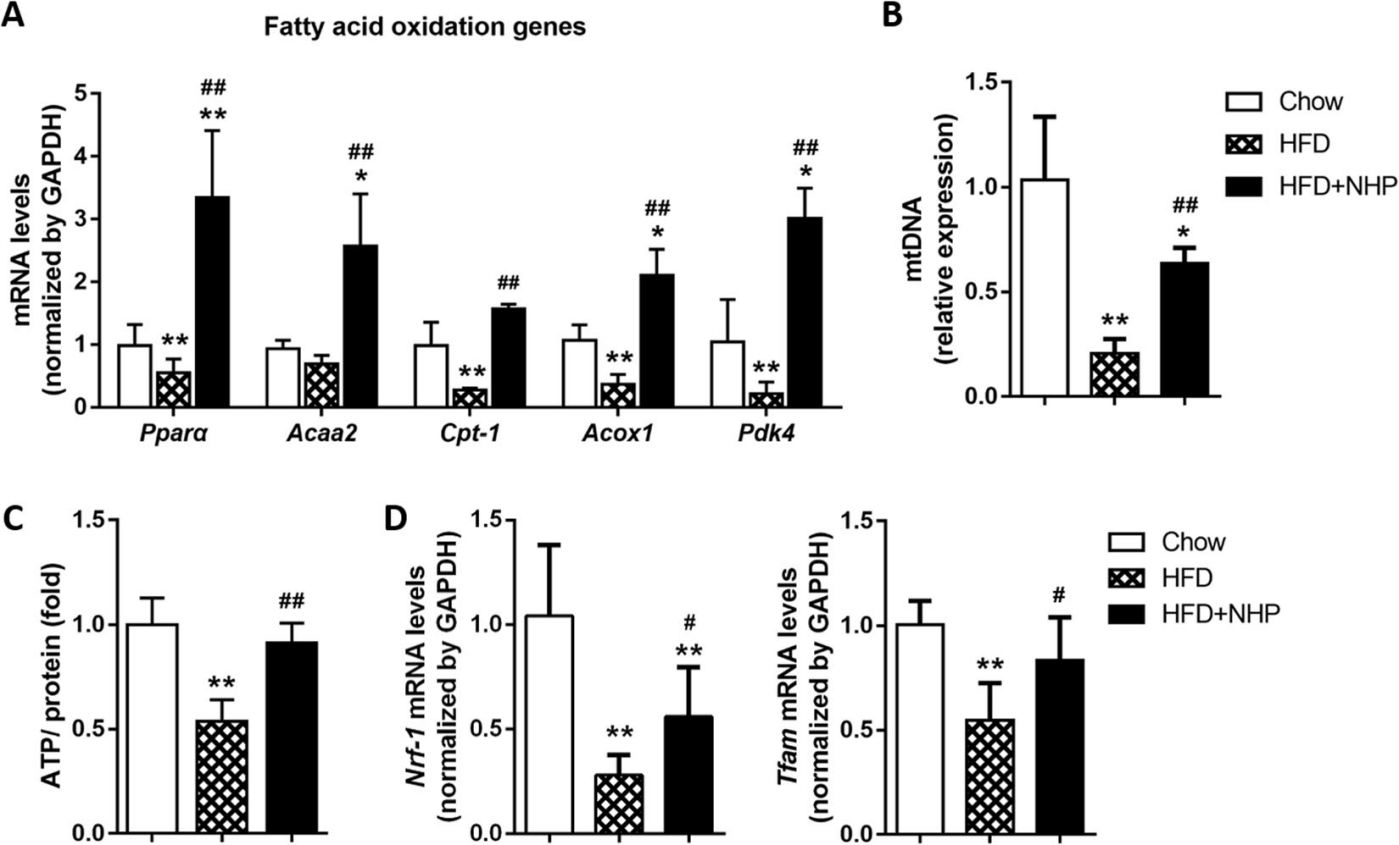
The effect of NHP on the fatty acid oxidation genes expression and mitochondrial biogenesis in liver of HFD-fed mice. **a** The mRNA expression of fatty acid oxidation genes was determined by real-time PCR. **b** Mitochondrial DNA content analyzed by quantitative PCR in liver from each group. **c** The ATP content was detected in liver. **d***Nrf-1* and *Tfam* mRNA analyzed by RT-PCR. All values were expressed as mean ± SD (*n* = 5). *p < 0.05, **p < 0.01, versus chow group; ^#^*p* < 0.05, ^##^*p* < 0.01, versus HFD group.

Under high-fat diets, increased lipid synthesis and reduced fatty acid oxidation promote hepatic steatosis^5,25^. We examined the expression of critical genes involved in fatty acid synthesis and fatty acid oxidation in liver. The result showed that NHP upregulated fatty acid oxidation gene expression, such as *Pparα, Acaa2, Cpt-1, Pdk4*, and *Acox1* (Fig. 4a), while downregulated the expression of lipogenic genes including *Srebf1*, *Fasn*, *Scd1*, and *Acc1* (Fig. S3) in the liver of HFD mice. Compared with fatty acid synthesis genes, NHP modulated fatty acid oxidation genes to a higher degree, which can be seen from the multiples of gene expression changes between HFD and HFD + NHP groups (Fig. 4a and S3).

### NHP elevates mitochondrial biogenesis in liver of HFD mice

In order to further clarify the mechanism by which NHP regulates fatty acid oxidation, we examined mitochondrial function in liver of HFD fed mice. Treatment with NHP markedly increased mtDNA copy number (Fig. 4b) and ATP content (Fig. 4c) in the liver of HFD mice, compared to their control counterparts. Of note, the expression of genes associated with mitochondrial biogenesis (*Nrf-1* and *Tfam*) was significantly up-regulated by NHP (Fig. 4d). These results suggested that the elevation fatty acid oxidation by NHP might be due to increased mitochondrial biogenesis in liver.

### NHP improves mitochondrial capacity by promoting PGC-1α expression

**Fig. 5:**
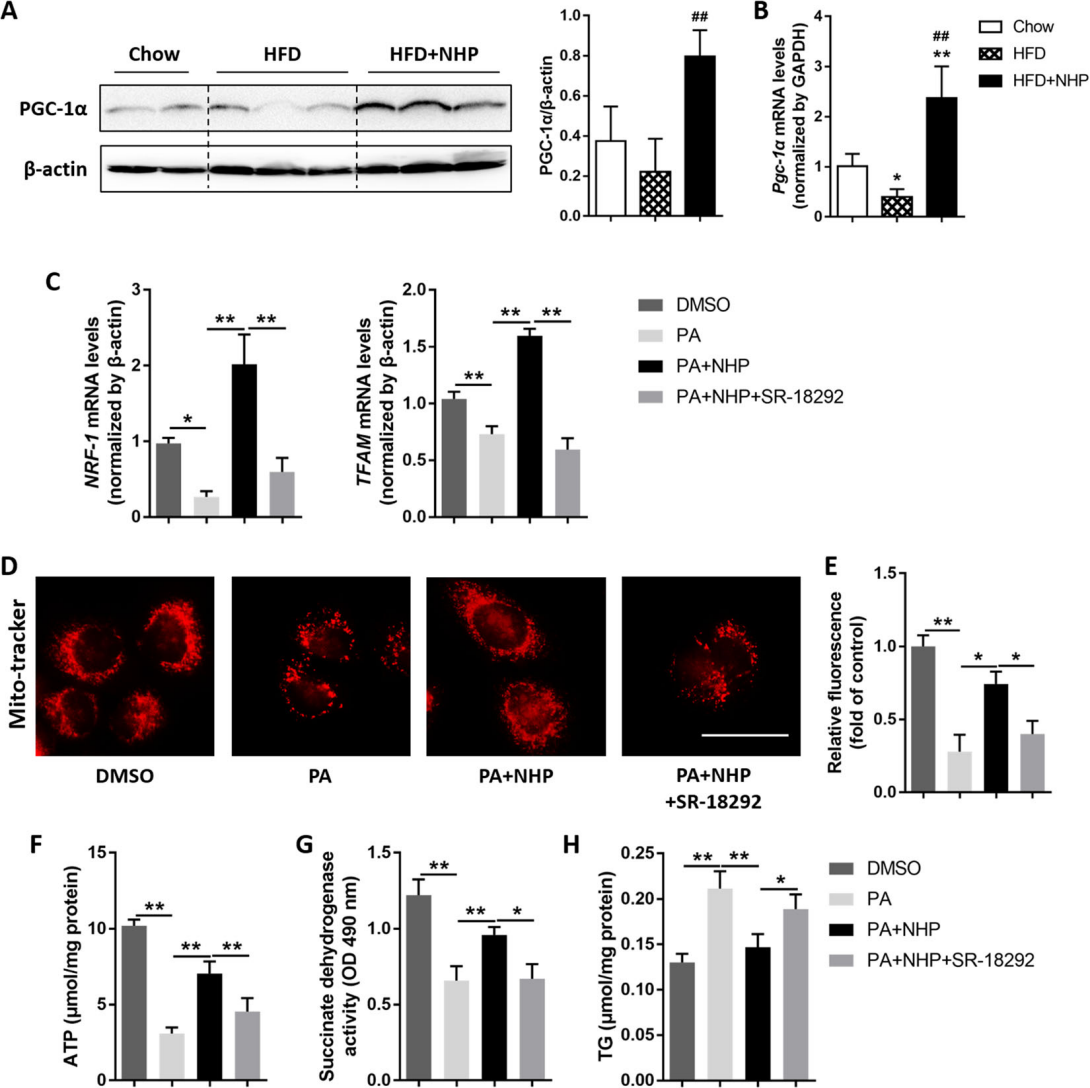
NHP promotes PGC-1α expression in vivo and improves PGC-1α-mediated mitochondrial biogenesis in vitro. **a** The protein level of PGC-1α in liver of HFD-induced mice was assessed by western blot analysis. The density of bands was normalized to β-actin. (**b**) The mRNA expression of *Pgc-1α* in liver of HFD-induced mice was determined by real-time PCR. Values were expressed as mean ± SD (*n* = 5). *p < 0.05, **p < 0.01, versus chow group; ^##^*p* < 0.01, versus HFD group. The HepG2 cells were treated with DMSO, 0.4 mM PA, 0.4 mM PA + 100 μM NHP or 0.4 mM PA + 100 μM NHP + 20 μM SR-18292 for 16 h, respectively, after starving in serum-free DMEM for 24 h. **c** The mRNA expressions of *NRF-1* and *TFAM* were determined by RT-PCR. Values were expressed as mean ± SD (*n* = 3). **d** The HepG2 cells were stained with Mito-Tracker (red) and visualized by fluorescence microscopy. Scale bar = 300 μm. **e** The fluorescence intensity (mitochondrial mass) was determined by Microplate reader. **f** The ATP content was detected in the HepG2 cells. **g** The level of succinate dehydrogenase in cellular mitochondria was determined using MTT. **h** The content of triglycerides (TG) in the HepG2 was assessed using the commercial kits. Values were expressed as mean ± SD (*n* = 6). *p < 0.05, **p < 0.01.

**Fig. 6:**
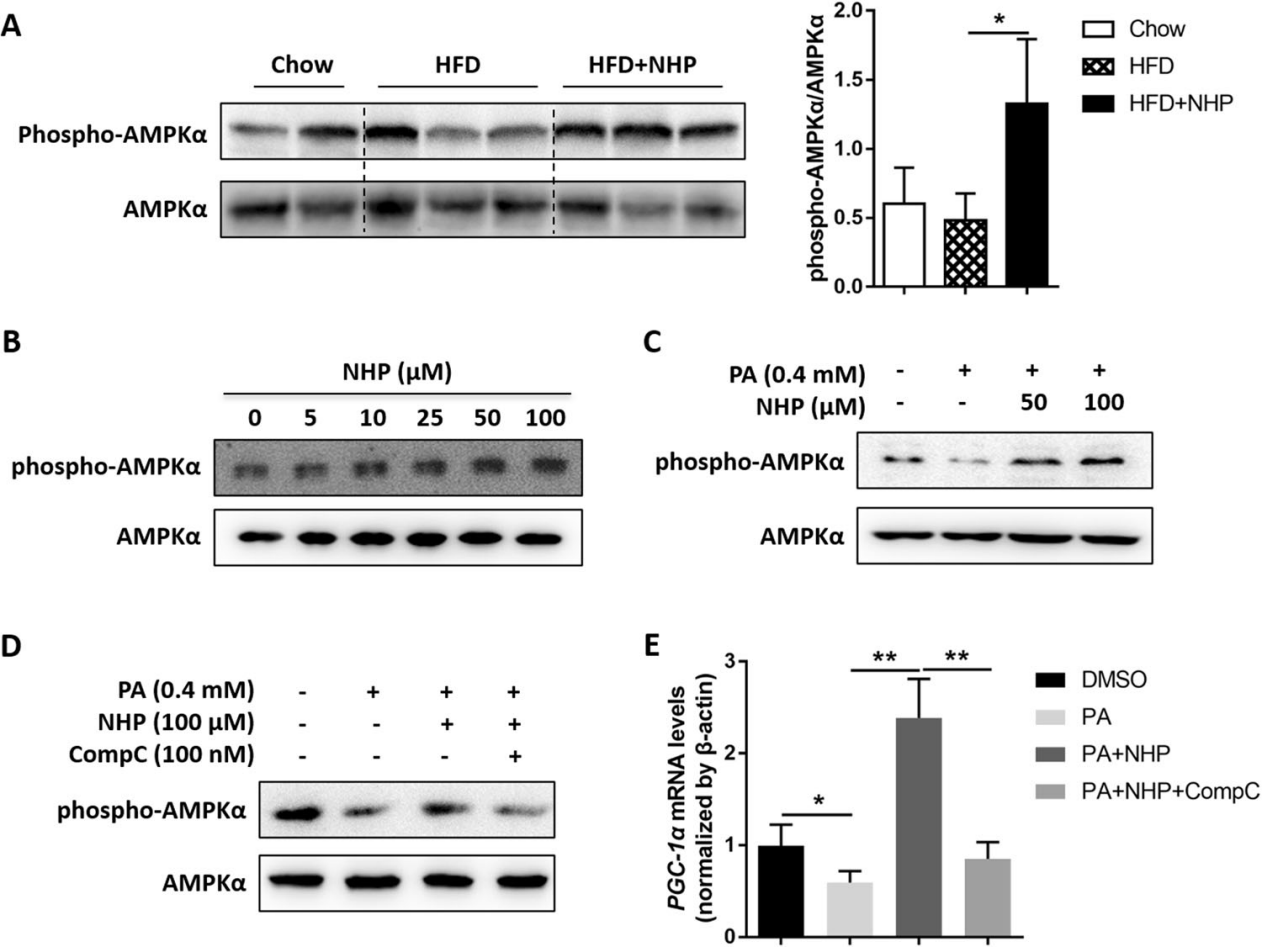
NHP stimulates the expression of PGC-1α by increasing AMPK activity. **a** The protein level of phospho-AMPK and AMPK in livers of HFD-induced mice were assessed by western blot analysis. The density of bands was normalized to AMPK. **b** The HepG2 cells were treated with various concentrations of NHP (0, 5, 10, 25, 50, 100 μM) for 16 h. The amount of phospho-AMPK and AMPK was measured by western blotting. **c** The HepG2 cells were treated with DMSO, 0.4 mM palmitate (PA), 0.4 mM PA + 50 μM NHP or 0.4 mM PA + 100 μM NHP for 16 h respectively, after starving in serum-free DMEM for 24 h. The amount of phospho-AMPK and AMPK was measured by western blotting. **d** The HepG2 cells were treated with DMSO, 0.4 mM PA, 0.4 mM PA + 100 μM NHP or 0.4 mM PA + 100 μM NHP + 100 nM Compound C (CompC) for 16 h, respectively. The amount of phospho-AMPK and AMPK was measured by western blotting. **e** The mRNA expression of *PGC-1α* was determined by real-time PCR. Values were expressed as mean ± SD (*n* = 3). *p < 0.05, **p < 0.01.

Since PGC-1α is a crucial coactivator for fatty acid oxidation and mitochondrial biogenesis^5,26^, we next examined its expression in the liver of HFD mice. The treatment of NHP markedly increased the protein abundance of PGC-1α in the liver of HFD mice (Fig. 5a). The mRNA expression of *Pgc-1α* was also upregulated by NHP (Fig. 5b). Palmitic acid (PA) incorporation in culture medium was previously reported to disrupt mitochondrial function and increase lipid accumulation in HepG2 cells^27,28^. To determine whether NHP increases mitochondrial biogenesis via upregulation of PGC-1α, we used SR-18292, a selective PGC-1α inhibitor, to block the transcriptional activity of PGC-1α^29^. As shown in Fig. 6c, administration of NHP obviously increased the mRNA expression *of* PGC-1α target genes, which was inhibited by PA in HepG2 cells. The addition of SR-18292 counteracted NHP-induced increase of *NRF-1* and *TFAM* (Fig. 5c). Mitochondrial mass is a crucial parameter for cellular oxidative capacity. *NRF-1* and *TFAM* are critical regulators for mitochondrial fragmentation and mass^30^. We examined the alteration of mitochondrial mass using Mito-Tracker Red in PA induced HepG2 cells treated with NHP alone or plus SR-18292. NHP treatment prominently increased PA induced reduction of mitochondrial mass, but it was largely decreased by SR-18292 (Fig. 5d, e). In line with this observation, SR-18292 also counteracted the increase of ATP generation and succinate dehydrogenase activity treated by NHP in PA-induced HepG2 cells (Fig. 5f, g), which were considered as important indicators for mitochondrial capacity^20^. Moreover, the reduction of TG level induced by NHP in PA treated HepG2 cells was also diminished by PGC-1α inhibition (Fig. 5h). These data suggested that NHP elevates mitochondrial biogenesis by promoting PGC-1α expression.

### NHP promotes PGC-1α expression by increasing AMPK activity

It has been shown that PGC-1α gene expression is induced by chemical activation of AMPK^31^. In this study, we showed that the phosphorylation of hepatic AMPK was consistently increased by NHP treatment in HFD-fed mice (Fig. 6a). Moreover, under regular culture condition, NHP stimulated AMPK phosphorylation in a dose-dependent manner in HepG2 cells (Fig. 6b). The addition of PA in culture medium downregulated AMPK phosphorylation while NHP treatment significantly reversed it (Fig. 6c). To examine whether AMPK is responsible for NHP induced PGC-1α, we re-inhibited increased AMPK activity by Compound C, a reversible AMPK inhibitor, after NHP treatment. We found that the mRNA of PGC-1α was obviously reduced after Compound C administration, compared to NHP treatment alone in PA-treated HepG2 cells (Fig. 6d, e). These results suggested that the increase of PGC-1α expression by NHP was via the simulation of AMPK activity.

## Discussion

Here, we identify, for the first time, that NHP has the effect on hepatic mitochondrial biogenesis in HFD-induced mice. We showed that NHP ameliorate hepatic steatosis and systematic insulin resistance in HFD fed mice. NHP elevated hepatic mitochondrial biogenesis and fatty acid oxidation by increasing PGC-1α expression. The promotion of PGC-1α expression by NHP was achieved by the activation of AMPK. Overall, PGC-1α-mediated mitochondrial biogenesis plays a vital role in the mitigating effect of NHP on hepatic steatosis.

Fatty acid oxidation, fatty acids uptake, de novo synthesis, and the secretion of very low density lipoprotein (VLDL) are the major pathways influencing liver lipid content^32,33^. Although the clinical significance of mitochondrial β-oxidative impairment in the progression of NAFLD is inconclusive, studies have shown that fatty acid oxidation plays an important role in reducing liver lipid accumulation^32^. Mice deficient in MCAD and VLCAD, the acyl-CoA dehydrogenases involved in the beta dehydrogenation of the acyl-CoA ester derived from fatty acid in the process of β-oxidation, develop hepatic steatosis even fed by chow diet, emphasizing the role of these proteins and fatty acid β-oxidation in the regulation of hepatic TG content^34^. Targeting peroxisomes, alternative organelles of nonmitochondrial compartments of the cell for fatty acid oxidation, either by hepatocyte-specific depletion of peroxisomes or by deficiency in ACOX (which catalyzes the initial step in peroxisomal fatty acid oxidation) expression results in hepatic lipid accumulation, arguing the role of fatty acid oxidation in NAFLD^35^. In our study, we found that NHP-induced fatty acid oxidation was a major way by which NHP improved fatty liver. This observation further supports that fatty acid oxidation has a beneficial effect on improving lipid accumulation in liver.

PGC-1α mediated mitochondrial biogenesis is essential for the enhancement of mitochondrial capacity and fatty acid oxidation^36^. PGC-1α acts as a cardinal transcriptional regulator of mitochondrial biogenesis by activating nuclear respiratory factor-1 (NRF-1), which induce the transcription of mitochondrial transcription factor A (TFAM) expression thereby upregulating mtDNA replication and transcription^26,37^. Besides, PGC-1α promotes fatty acid β-oxidation by acting as a co-activator of PPARα, which in turn promotes the expression of genes related to mitochondrial fatty acid catabolism^38^. In the present study, we confirm that the increase of PGC-1α is required for mitochondrial biogenesis after NHP treatment.

Increased fatty acid oxidation not only reduces the lipid overload and lipotoxicity under HFD, but also produces ROS. Excessive fatty acid oxidation may overwhelm the capacity of the antioxidant defense system and induce oxidative stress and hepatic inflammation^39^. Intriguingly, NHP repressed oxidative stress and inflammatory response in liver induced by HFD, counteracting the adverse effect of enhanced fatty acid oxidation. In addition to the role on mitochondrial biogenesis, PGC-1α is also reported to be a regulator of antioxidant enzymes in response to oxidative stress^40,41^. PGC-1α reduces mitochondrial ROS production through upregulation of antioxidant genes expression such as *Cat*, *Sod1*, *Gpx1*, and *Ucp2*^41^. In current study, we find that the expression of these antioxidant genes is elevated by NHP in vivo. Overproduction of ROS can lead to mitochondrial damage, including mutations in mitochondrial DNA, damage to the mitochondrial respiratory chain and mitochondrial membrane permeability^42^. We speculate the elevation of mitochondrial capacity is at least partially due to the relief of oxidative stress by NHP treatment, and the up-regulation of PGC-1α promoted antioxidant enzyme expression could be one of the underlying mechanisms.

The energy sensor AMPK is a master regulator in the control of energy metabolism in liver. At the posttranslational level, AMPK could activate PGC-1α by direct phosphorylation of its Ser^538^ residue^31^ or indirectly- by activating NAD^+^-dependent deacetylase SIRT1^43^. At the transcriptional level, AMPK also promotes PGC-1α expression^41,44^. AMPK activator, 5-aminoimidazole-4-carboxamide-1-b-d-ribofuranoside (AICAR), induces PGC-1α transcription in rat muscles^45^. Additionally, metformin increases PGC-1α mRNA and protein expression via AMPK dependent manner in primary hepatocytes^46^. In this study, we find that NHP elevates the level of PGC-1α is also AMPK dependent.

Taken together, our results demonstrate that NHP alleviates hepatic steatosis and insulin resistance in HFD fed mice. NHP increases hapatic mitochondrial biogenesis by promoting PGC-1α expression. The activation of AMPK could be the underlying mechanism through which NHP regulates PGC-1α expression. This suggests that NHP has the potential to become a dietary supplement for the auxiliary treatment of NAFLD.

## Supplementary information

Supplementary Figures

Supplementary Fig. S1

Supplementary Fig. S2

Supplementary Fig. S3
