# Supplementary material for: Neohesperidin enhances PGC-1α-mediated mitochondrial biogenesis and alleviates hepatic steatosis in high fat diet fed mice
Source: Nutr Diabetes. 2020 Aug 5;10:27. doi: 10.1038/s41387-020-00130-3 (PMC7406515; doi:10.1038/s41387-020-00130-3)
Supplement: Supplementary file 1 — Supplementary Figures [file 41387_2020_130_MOESM1_ESM.docx]

**Supplemental Fig. S1 The effect of NHP administration on physiological indices in HFD-induced mice.** C57BL/6 mice were fed either a chow or a high fat diet (HFD) for 12 weeks. Mice were treated with daily oral doses of NHP (50 mg/kg). Water was gavaged as control. **(A)** Food intake. **(B)** Body weight gain. **(C)** Epididymal adipose weight. Data were expressed as the mean ± SD (n=12). ^*^*p*<0.05, ^**^*p*<0.01, versus chow group; ^#^*p*<0.05, ^##^*p*<0.01, versus HFD group; N.S., no significance.

**Supplemental Fig. S2 The effect of NHP on systematic insulin resistence in HFD-fed mice. (A)** Fasting blood glucose (FBG) level in last week. **(B)** Fasting insulin (FINS) level. **(C)** Homeostasis model assessment of insulin resistance (HOMA-IR) index. Data were expressed as the mean ± SD (n=12). **(D)** Oral glucose tolerance test (OGTT, 2 g/kg body weight, p.o.) in each group mice at week 8. **(E)** Insulin tolerance test (ITT, 0.75 U/kg body weight, i.p.) in each group mice at week 8. Data were expressed as the mean ± SD (n=5). ^*^*p*<0.05, ^**^*p*<0.01, versus chow group; ^#^*p*<0.05, ^##^*p*<0.01, versus HFD group.

**Supplemental Fig. S3 NHP suppresses the mRNA expression of lipogenesis genes in liver of HFD-induced mice.** The mRNA expressions of *Srebf1*, *Fasn*, *Scd1* and *Acc1* were determined by RT-PCR. Values were expressed as mean ± SD (n=5). ^*^*p*<0.05, ^**^*p*<0.01, versus chow group; ^#^*p*<0.05, ^##^*p*<0.01, versus HFD group.
