# Supplementary figures and images for: Neohesperidin enhances PGC-1α-mediated mitochondrial biogenesis and alleviates hepatic steatosis in high fat diet fed mice
Source: Nutr Diabetes. 2020 Aug 5;10:27. doi: 10.1038/s41387-020-00130-3 (PMC7406515; doi:10.1038/s41387-020-00130-3)

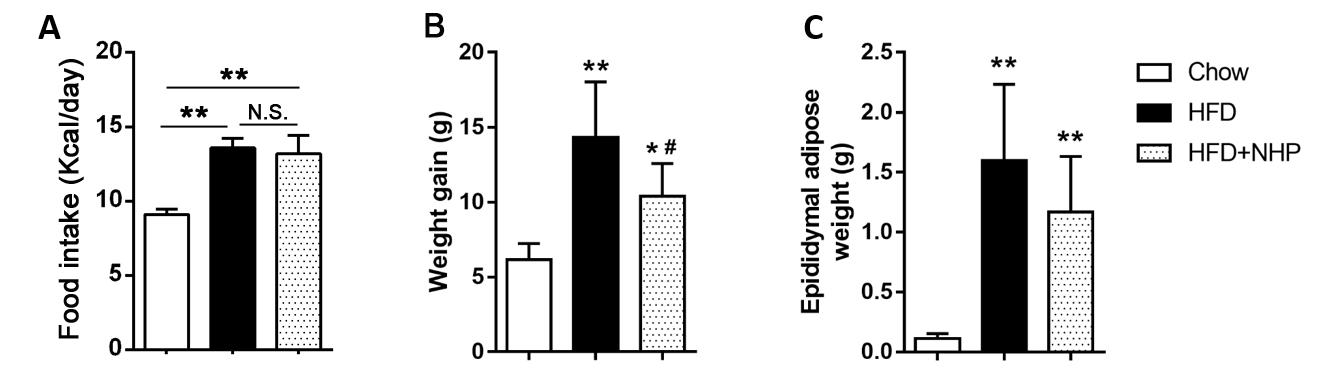

Supplement: Supplementary file 2 — Supplementary Fig. S1 [file 41387_2020_130_MOESM2_ESM.tif]

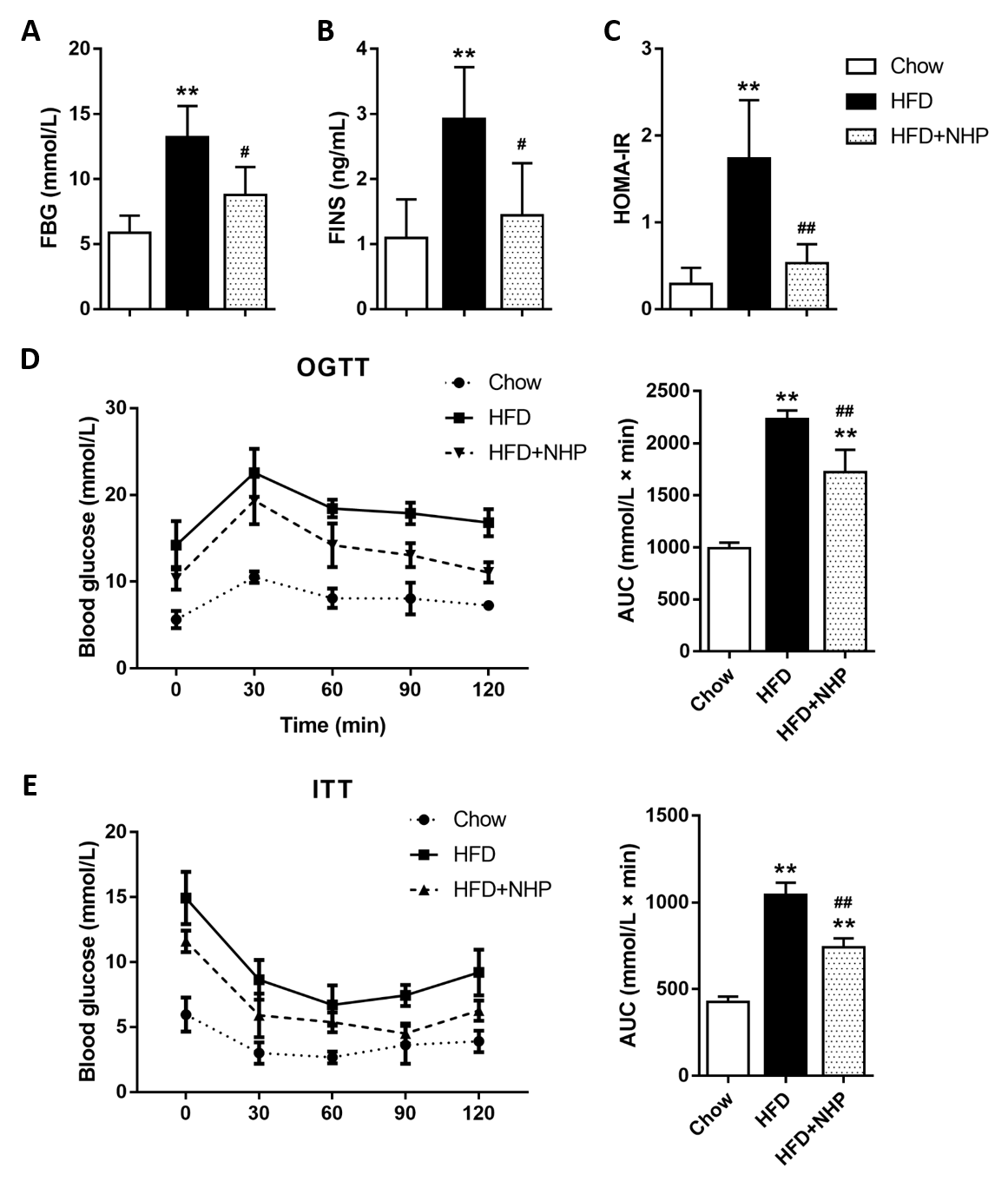

Supplement: Supplementary file 3 — Supplementary Fig. S2 [file 41387_2020_130_MOESM3_ESM.tif]

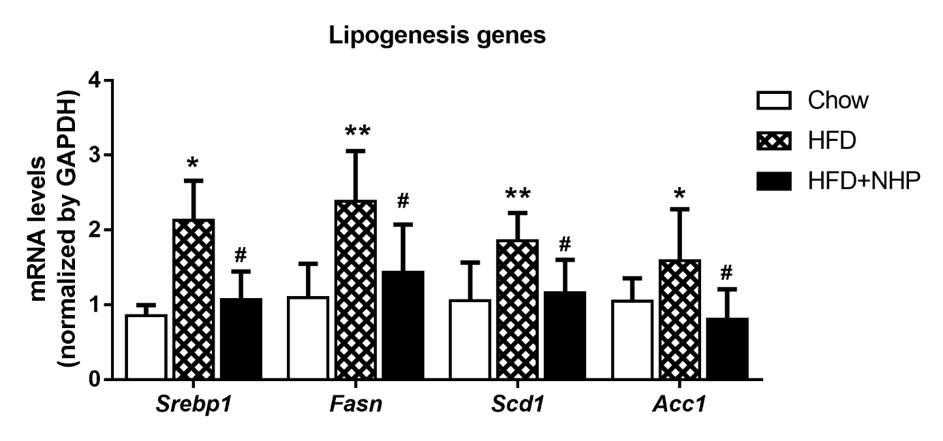

Supplement: Supplementary file 4 — Supplementary Fig. S3 [file 41387_2020_130_MOESM4_ESM.tif]
